# Framing access to essential medicines in the context of Universal Health Coverage: a critical analysis of health sector strategic plans from eight countries in the WHO African region

**DOI:** 10.1186/s12913-022-08791-9

**Published:** 2022-11-22

**Authors:** Alison T. Mhazo, Charles C. Maponga

**Affiliations:** 1 Ministry of Health, Community Health Sciences Unit (CHSU), Private Bag 65, Area 3, Lilongwe, Malawi; 2 Department of Pharmacy and Pharmaceutical Sciences, Faculty of Medicine and Health Sciences, University of Zimbabwe, Avondale, P. O. Box A178, Harare, Zimbabwe

**Keywords:** Framing, Access to essential medicines, WHO African Region, National strategic plan

## Abstract

**Background:**

Framing affects how issues are understood and portrayed. This profoundly shapes the construction of social problems and how policy options are considered. While access to essential medicines (ATM) in the World Health Organization (WHO) African Region is often framed as a societal problem, there is dominance of medical and technically oriented approaches to analyze and remedy the situation. Hence, the systematic application of social science approaches, such as framing theory, remains under-explored. Through a framing analysis of National Strategic Plans (NSPs) from eight countries, this study explores the applicability and potential usefulness of framing theory to analyze essential medicines policies.

**Methods:**

We inductively coded the relevant NSP textual fragments using the qualitative content analysis software ATLAS.ti.22. Benford and Snow’s conceptualization of framing was used to organize the coded data into three frames: diagnostic (problems), prognostic (solutions) and motivational (values and ideological).

**Results:**

The following five diagnostic frames were dominant or in-frame: medicine unavailability, ineffective regulation, weak supply chain management, proliferation of counterfeit (substandard or falsified) medicines and use of poor quality medicines. Diagnostic frames related to financing, affordability, efficiency and corruption were given limited coverage or out of frame. Prognostic frames corresponded with how these problems were framed. Whilst Universal Health Coverage (UHC) and its guiding principles was the dominant motivational frame, we identified some frame discordance between the global discourse and national level policies.

**Conclusions:**

Social science approaches such as framing analysis are applicable and useful to systematically analyze essential medicine aspects. By applying framing theory, we revealed that ATM aspects in the eight countries we analyzed are more often characterized in relation to availability at the expense of affordability which undermines UHC. We conclude that whilst UHC is a strong motivational frame to guide ATM aspects, it is insufficient to inform a comprehensive approach to address the problems related to ATM at country level. To effectively advance ATM, concerned actors need to realize such limitation and endeavor to gain a deeper understanding of how problems are framed and agendas are set at country level, the processes through which ideas and knowledge become policies, including the political demands, incentives and trade-offs facing decision-makers in selecting policy priorities.

**Supplementary Information:**

The online version contains supplementary material available at 10.1186/s12913-022-08791-9.

## Background

Equitable access to essential medicines has been recognized as a key component for achieving universal health care for the past fifty years [1–4]. As a result, the quest to achieve universal access to essential medicines (ATM) has prominently featured in all the major global health discourses from pioneering concepts such as Primary Health Care (PHC) concept to contemporary Universal Health coverage (UHC). The quest to ensure access to essential medicines is not just an aspirational rhetoric, for the discovery of modern medicines has dramatically altered the course of disease control and alleviated human suffering in the past century. Historical precedents loom large, from the breakthrough discovery of penicillin by Alexander Fleming in 1928 which significantly improved the management of sepsis and became part of the World War 11 machinery [5]. In the 1940s, the discovery and use of effective medicines for malaria and tuberculosis is credited for reversing the devastating effects of these diseases whilst recent memory vividly captures how anti-retroviral medicines transformed HIV/AIDS from a ‘death sentence into a chronic, but treatable disease’ [6–8]. Thus, the discovery of medicines has been a central symbol of ingenuity and dedication to alleviate human suffering and preserve longevity.

Despite their utmost importance in influencing health, access to essential medicines remains one of the most pressing global challenge and WHO estimates that two billion people all over the world do not have sufficient access to essential medicines [9, 10]. The foregoing account therefore reveals one of the most challenging paradox in global health -the uttermost need for essential medicines and their widespread inaccessibility in reality,  particularly in Low and Middle -Income countries (LMICs). This situation has been attributed to various factors amongst them patent systems that favor profits over public health [8, 11–13], weak health systems [14, 15] and widespread corruption [16, 17].

Lack of access to essential medicines also reveals pervasive global inequities [4, 18], albeit with minimal political action to remedy the situation [19]. Inequitable access to medicines has been attributed to the global politics of pharmaceutical monopoly [20, 21] which in itself is a reflection of the structural global power asymmetry between industrialized and non-industrialized countries. This global power asymmetry has been a persistent problem since at least the 1970s [1, 22] and again has been laid bare by the current skewed global distribution of the COVID-19 vaccine [23, 24]. The framing of inequitable access to essential medicines as something ‘man-made’ to maximize profits at the expense of human suffering has evoked some deep moral, morally inclined convictions over the matter. As a result, actors concerned with access to medicines have advocated for a ‘fairer’ system based on the principles of human rights, social justice and solidarity [19, 25, 26]. The pursuit of equitable access to essential medicines sits very well with the aspiration of UHC-a principle that asserts that individuals should get access to health care without facing financial hardship [4]. In this vein, there has been a concerted global effort to elucidate the problem of inadequate access to medicines and propose solutions to address the identified problems.

This process of defining problems-their scope, causes and who is affected-and the subsequent attachment of solutions to address those problems is referred to as issue framing [27]. Despite the existence of globally articulated problems and proposed solutions to promote access to medicines in developing countries, little attention has been paid to what problems get prioritized at country level; the related solutions and its potential effect on UHC; aspects that are all underpinned by the way an issue is framed. In this study, we sought to understand the applicability and usefulness of framing theory to analyze access to medicines policies through a review of health sector strategic plans from eight countries in the WHO African region. Whilst there is global literature that mainly focuses on the trade related power contestations in relation to access to medicines particularly at agenda setting stage [23, 26, 28, 29], we decided to focus on the official policy content at country level, which reflects what governments prioritizes for implementation. It is however important to mention upfront that the aim of this analysis is neither to rate the national strategic plans (NSPs) nor to comparatively benchmark them, but to better understand how the issue of ensuring access to essential medicines is framed at the national level across countries and what, if any, impact this seems to have on the solutions proposed.

The remainder of the paper proceeds as follows. First, we present framing theory and apply it to the evolution of essential medicine policies. We then describe the methods followed by the presentation of findings. The findings are then discussed in relation to their implications on UHC in the selected countries and their potential usefulness in other settings before we conclude.

### Framing theory

Framing theory focuses on the primacy of ideas in explaining policy dynamics in contrast to other theories that are oriented towards interests and institutions [30]. Ideas have been known to be powerful in shaping social preferences as described by political scientist Deborah Stone ‘ideas are a medium of exchange and a mode of influence even more powerful than money, votes and guns’. Shared meanings motivate people into collective action whilst divergent ideas  are  at the center of all political conflict. Thefore, policymaking, is a constant struggle over the criteria for classification; the boundaries of categories, and the definition of ideals that guide the way people behave’’ [31]. At the core of ideational policy making is the construction of social problems. Frames are therefore powerful because they shape how problems are perceived by excluding or emphasizing particular information. In this context, framing is a meaning-making process that portrays an issue in a particular way which determines what is termed a routine condition that can be tolerated or a social problem that requires policy action [32]. The process of framing typically has four components namely a defined problem, causal agents of the problem, judgement of the causal agents and their effects and suggested solutions [33]. Theoretically, framing analysis draws from various disciplines spanning psychology, political science, communication and social movement.

Problem framing also determines who gets involved. Issues can be framed to make them appear technical and only relevant to technical aspects or linked to wider societal values to heighten participation [27]. As a result, individual actors involved in policy process constantly re-frame problems to influence policy portrayals and increase the number of people mobilized around an issue [34], a process known as conflict expansion [35]. Conflict expansion facilitates the shifting of policy venues or ‘the institutional locations where authoritative decisions are made concerning a given issue’ [36]. Through strategic framing, policy makers constantly engage in ‘venue shopping’ which involves the recruitment of previously uninterested stakeholders [35]. An example of the effect of conflict expansion and venue shopping is in relation to the global access to anti-retroviral (ARVs). By framing access to HIV/AIDS treatment as a human right [12], provision of ARVs became an issue of a wider societal concern that mobilized the interest of activists and pressure groups [37]. Empowered by the discursive framing of human rights and social justice, these interest groups shifted the venue to the political and pharmaceutical industry arena to lobby for radical reforms in patent regimes including the use of compulsory licensing [38]. It is also important to note that whilst policy frames can increase mobilization around an issue, they can also be designed to de-mobilize interest.

In this paper, we use the Benford and Snow’s conceptualization of frames, which is influenced by the social movement paradigm [39], for several reasons. First, according to Benford and Snow, framing is concerned with cognitive mechanisms by which grievances are interpreted, given direction and consensus around the goals of reform. We found this characterization to be consistent with the nature of essential medicines. As stated earlier, the history of essential medicines can be viewed as a long-drawn, grievance driven arena around equitable access to medicines under which there has been intellectual and social movement to address those inequities. Second, in line with the social movement lens, there has been an enduring effort to give direction and consensus to the organization of provision of medicines since the emergence of the essential medicines concept in the mid-1970s. Benford and Snow (1988) argued that any social movement actor involved in mobilization and therefore the attempt to move people “from the balcony to the barricades” on a particular issue has to attend to three “core framing tasks”—“diagnostic,” “prognostic,” and “motivational” framing [40]. These core-framing tasks are followed by specific problem-setting stories. Table 1 below shows Benford and Snow’s concepts of framing.

**Table 1:** Benford and Snow’s concepts of framing

Framing tasks	Diagnostic framing	What is the problem? How is it defined?
	Prognostic framing	How do we solve the problem?
	Motivational framing	How do we argue for our definitions and solutions – ideology

Diagnostic framing involves the identification of a problem and the attribution of blame or causality while prognostic framing is concerned with the proposed solution to the identified problem and the related indication of strategies, tactics, and goals. On the other hand, motivational framing justifies the rationale for action. Diagnostic and prognostic framing is geared towards “consensus mobilization”— creating a shared picture of problem and solution—while motivational framing is aimed at “action mobilization,” pushing collective action on the basis of shared perceptions [40].

### Framing with respect to essential medicines

In relation to access to essential medicines, various diagnostic, prognostic and motivational frames exist at global level; often coinciding with the ushering of major global discourses such as Primary Health Care (PHC) [41] and Universal Health Coverage (UHC) [42].

### Diagnostic framing

In the mid-late 1970s, the dominant diagnostic frame was the proliferation of medicines that did not align with the priority health needs of the population and general lack of access to medicines particularly in developing countries [43]. Attendant problems included unethical promotion of medicines by manufacturers and high pricing. At the inception of PHC and the essential medicines concept in developing countries, the framing shifted towards more technical aspects such as reliable supply chain systems, financing and rational use of medicines within the framework of national drug policies [3]. In the 1990s, the framing was dominated by the frame misalignment between trade related aspects and access to HIV/AIDS treatment [38]. The 2000s coincided with diagnostic frames that rose to the agenda within the UHC discourse. These include unaffordability, reliance on out of pocket expenditure, sub-optimal quality and safety, irrational use of medicines  and limited investment in developing medicines for patient populations that do not represent a profitable market [4]. Under UHC, the other issue that has emerged as a major problem relates to corruption and wastage in the pharmaceutical sector [42].

### Prognostic framing

In line with Benford and Snow’s conceptualization of framing, the prognostic frame has followed the diagnostic frame in relation to ATM. To address the pervasive proliferation of inappropriate medicines, the WHO adopted the essential medicines concept and developed the first model list of essential drugs in 1977 to provide ‘drugs that satisfy the health care needs of the majority of the population’ [44]. This prognostic shift has been dubbed ‘a peaceful revolution in international public health’ [18] to reflect its enduring influence on how ATM is conceived globally. In the aftermath of international acceptance, the prognostic frame shifted towards the diffusion of the concept to country level. In 1982 the WHO launched the essential drugs Action Programme to guide the translation of the essential medicines concept into country level policy. In line with the problems encountered at country level, the prognostic frame emphasized technically oriented interventions such as strengthening supply chains, promoting rational medicines use and ensuring sustainable financing. The prognostic frame of the 1990s is dominated with balancing the conflicting interest of trade and public health in light of the HIV/AIDS epidemic.  The 2000s is anchored on ensuring access to essential medicines without financial hardship in line with UHC whilst ensuring efficiency. Consequently, the prognostic frame under UHC emphasizes effiency enhancing strategies , including  initiatives aimed at curbing corruption in the pharmaceutical sector such as the WHO’s Good Governance for Medicines [16, 17, 45, 46].

### Motivational framing

Throughout the years, advancing ATM has been driven by underlying ideological frames that are paired with public health concerns. In the 1970s, the discursive motivational framing for advancing ATM was rooted in the discourse of decolonization and global solidarity under PHC [3]. The 1990s is dominated by the human rights and social justice oriented approach in light of the HIV/AIDS epidemic. ATM under the contemporary UHC discourse is driven by the need for equity which shaped by a recombination of previous motivations for global solidarity, human rights and social justice [4, 25, 47].

## Methods

Below we present the data collection process, selection criteria and data analysis.

### Data collection

Between July and November 2021, we searched the internet for publicly available NSPs from African countries by typing the name of the country combined with terms such as health strategic plan or national health strategy. We opted for NSPs for several reasons. First, NSPs are official documents that carry significant legitimacy in guiding the short and long-term plans of a country including high level endorsement at the level of the Minister of Health. Second, NSPs  are designed and formulated by a range of in-country stakeholders and international partners from which there is a presumption that they capture varied actor interests. Third, NSPs contain a snapshot of all health priorities which allows a rapid comparison of the issue of interest (in our case access to medicines) and other areas in a particular setting. In this regard, they facilitate a ‘one stop shop’ review that may not be feasible if specific and numerous guidelines would be obtained for all health priorities. Finally, we opted for NSPs instead of national medicines policies since the latter tends to be too broad, aspirational, long term and infrequently updated. Conceptually, NSPs represent a good source for policy analysis because  government has been generally viewed as a predominantly text based medium where official documents provide valuable insights regarding policy positions [48]. Thus, document analysis has been promoted as a potent analytical approach in health policy research [49] which has been at the center of unpacking how governments frame intractable policy issues [48, 50, 51].

### Selection criteria

The initial selection of the countries was influenced by two main factors: 1) availability of the NSP from publicly available internet website sources. The exception was for Zimbabwe where the NSP was newly released and not yet uploaded on internet sites and the authors had to access the document through own network; 2) presentation of the NSP in English Language. Using this initial criteria, NSPs were retrieved from thirteen African countries: Malawi, Zimbabwe, Zambia, South Africa, Uganda, Tanzania, Kenya, Rwanda, Ghana, Ethiopia, Nigeria, Lesotho and Cameron. Out of the initially selected thirteen NSPs, those from five countries were excluded: Lesotho, Uganda, Ghana, Ethiopia and Malawi. The reason for exclusion by country were as follows: Lesotho (only a draft version could be retrieved), Uganda (retrieved NSP lapsed in 2020), Ethiopia (retrieved NSP lapsed in 2020), Ghana (medium term plan which lapsed in 2017 could be retrieved) and Malawi (though the NSP lapses in 2022 the authors were aware of the on-going effort for a successor plan where the medicines component is expected to be significantly transformed). Table 2 below provides a summary of the selection criteria.

**Table 2:** Included and excluded countries

Initial list of countries	Excluded countries	Final list of countries with reviewed NSPs
Malawi, Zimbabwe, Zambia, South Africa, Lesotho, Uganda, Tanzania, Kenya, Rwanda, Ghana, Ethiopia, Nigeria, Cameron, Ethiopia	Lesotho, Uganda, Ghana Malawi andEthiopia	Zimbabwe, Zambia, South Africa, Tanzania, Kenya, Rwanda, Nigeria and Cameron

From a feasibility and data analysis standpoint, the eight countries represented a limited number of NSPs to allow a detailed analysis of the strategic plans yet maintaining some degree of geographical representation of the WHO African regions. The relevant details and links to the full versions of the analyzed documents (NSPs) can be found in Additional file 1.

### Data analysis

The entire NSP text was uploaded in the qualitative analysis software ATLAS.ti.22. One author conducted an inductive frame analysis by closely reading the NSP and coding the relevant textual fragments. Since the analysis was inductive, the codes were developed during the analysis. Every text that identified a problem related to ATM was coded with ‘diag’ to imply the diagnostic frame including repeat mentions of the same problem. This included text that mentioned challenges, gaps, bottlenecks and weaknesses. Similarly, every text that identified a solution related to ATM was coded with ‘prog’ to imply the prognostic frame. This included text that mentioned strategies, interventions, aims and action plans. A code-document table was generated in ATLAS.ti.22. to show the total relative frequencies or mentions of each diagnostic and prognostic frame. Since motivational framing is ideological (shaped by societal meanings, values and beliefs) and therefore not specific to a technical domain such as ATM, we derived the relevant frames from the introduction, executive summary or preamble of the whole NSP. Methodologically, instead of coding the motivational frames in ATLAS.ti.22, we extracted the vision, mission and guiding principles verbatim from the NSPs.

### Study strengths and limitations

This study has limitations and strengths that are worthy acknowledging.

### Limitations of the study

Methodologically, in terms of data sources the findings of the study solely relied on NSPs that could be retrieved from internet searches. Therefore, some countries may have been declared as having lapsed NSPs when in reality they have updated versions that we could not retrieve at the point of searching. Second, by relying on the NSPs, it is possible that certain aspects could have been missed in the NSPs but covered in other policies and guidelines. We however posit that the NSP is an authoritative document that captures the main priorities of the country’s health sector and the aspects missed could be a sign of lower priority attached to them. Finally, this study is purely a content analysis and does not reflect how frame contestations influenced what was eventually incorporated into the NSPs, including the role of power which is a matter of central concern in policy making. In terms of generalizability, although the study drew from the widest universe of cases according to our inclusion and exclusion criteria, we could only analyze eight NSPS which potentially limits the scope of generalizability to other countries. Furthermore, only NSPs written in English could be analyzed which limits generalization to non-English speaking countries. Although the sample size was small, the NSPs that were ultimately selected represent a regional balance across Southern, Eastern and Western Africa with a range from low to middle income countries. However, it is important to note that whilst generalizability is a desirable study attribute, it is not naturally expected for qualitative studies that tend to be context specific [52]. Nonetheless, there are analytical and theoretical aspects that make our findings amenable to generalization. From an analytical standpoint, our findings can be generalizable in other African countries from the proximal similarity model, where generalizability of one study to another is judged by similarities between the time, place, people and other social contexts [53]. In this context, generalizability can arise from the similarities of African countries in terms of low socio-economic status, pervasive lack of access to essential medicines and the continent-wide pursuit of UHC. Theoretically, our findings can be generalizable from an interpretivist tradition which seeks to predict a theoretical understanding of the topic under examination [54]. In this case, generalization is interpreted as generalization towards a theory, rather than towards the population as the case with quantitative studies [55]. In terms of study repeatability, whilst we coded diagnostic and prognostic frames with the aid of ATLAS.ti.22 , the motivational frame was coded from a simple textual reading of the NSPs since there was no variation of interest across all the countries with convergence on UHC. If the study is to be repeated amongst countries where there is variation across all the frames, researchers should consider a uniform coding process.

### Strengths of the study

To our knowledge, this is the first study to apply framing theory to analyze NSP content on ATM and its implication on UHC in LMICs. This marks an important departure from the traditional norm of technically oriented analysis for essential medicines policies. From an interpretivist perspective described above, our study points to the wider applicability of social science approaches such as framing theory to analyze essential medicine policies in sub-Saharan Africa. Second, our findings are drawn from very recent sources that overlap in time which facilitates comparison.

### Findings

Below we present the framing of access to medicines in the context of UHC at national level (national strategic plans).

### Diagnostic framing

**Fig. 1:**
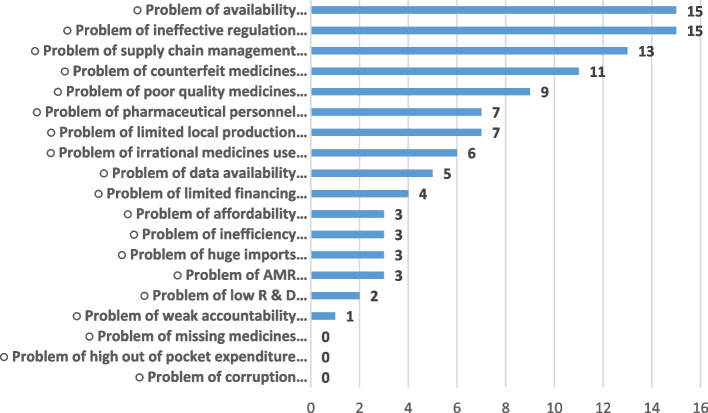
Diagnostic framing (frequency of problem mention)

Figure 1 below shows the frequency of problems cited in the NSPs.

### In –frame and out -of frame problems

Nineteen [19] diagnostic frames (problems) were identified from the coding process with a total of 107 mentions. Out of these, five [5] frames dominated: 1) Problem of availability 2) Problem of ineffective regulation 3) Problems of weak supply chain management 4) problem of substandard and falsified medicines 5) Problem of poor quality medicines. Out of the nineteen diagnostic frames, the problem of missing medicines, high out of pocket (OOP) expenditure and corruption did not specifically appear in any of the strategic plans. Table 3 below summarizes the top 5 (in-frame) and bottom 5 (out -of- frame) problems.

**Table 3:** Summary of top five in –frame and out -of frame problems

Top 5 mentioned problems (in-frame issues)	Bottom 5 mentioned problems (Out -of -frame issues)
Availability of medicines	Corruption
Procurement and supply chain management	Pricing/High Out of Pocket expenditure
Regulatory weakness	Missing medicnes
Product quality	Weak accountability
Fasified and substandard medicines	Low R & D

### Prognostic framing

**Fig. 2:**
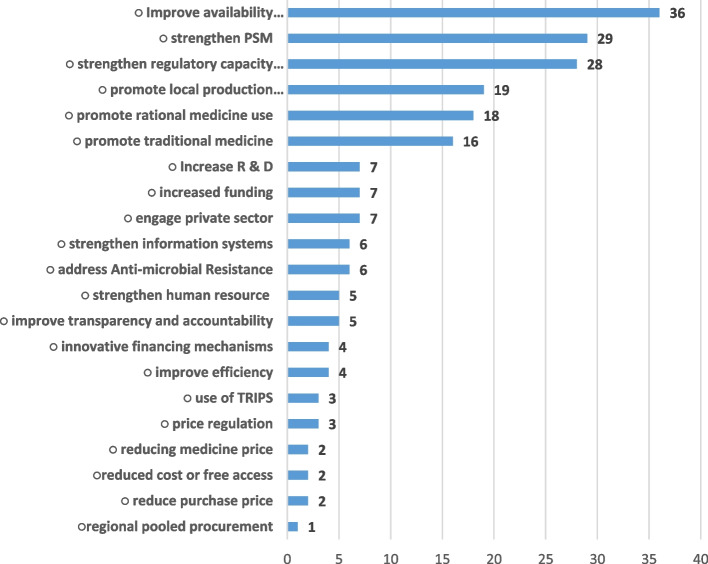
Prognostic framing (frequency of solution mention)

Figure 2 below shows the frequency of interventions or stragegies to address the problems.

Twenty-one [21] prognostic frames (interventions to address problems) were identified from the coding process with a total of 210 mentions. Consistent with framing theory, the prognostic frames generally followed the diagnostic frames. Interventions directly aimed at improving medicine availability dominated the diagnostic framing; accounting for 36 out of the 210 mentions. Other dominant prognostic frames included strengthening of procurement and supply chain management (PSM) and strengthening of regulatory systems. Prognostic frames that received the least mentions include those aimed at financing mechanisms, addressing inefficiencies, use of Trade-Related (TRIPS) flexibilities, reducing the purchase price at central (national) procurement level, price regulation at consumer level and regional pooled procurement mechanisms. Prognostic frames were also not evenly distributed across countries, particularly for the frames with lower coverage. In relation to price regulation, all three prognostic frames that explicitly mentioned the strategy were from Cameroon including past and planned efforts for price harmonization between the private and public sector. The four prognostic frames on innovative financing systems came from Cameroon, Kenya and Nigeria. Rwanda is the only country that explictly mentioned use of pooled procurement mechanism as a prognostic frame in addressing some of the pharmaceutical sector challenges.

### Motivational framing

All countries cited UHC as the foundational and overarching ideological frame to anchor the priorities for the health sector; often carved within long term developmental aspirations. Recurring guiding principles across countries include equity, quality, accountability and efficiency. Overall, there is not a lot of variation in the findings that is of interest in relation to motivational framing and this has been presented as an appendix (see Additional file 2).

## Discussion

This section discusses the findings of the study and its implications in relation to ATM in the context of UHC.

### In-frame and out-frame access to essential medicines issues and implications for UHC

In the eight countries studied, the main issues given attention (in-frame) in the NSPs are on availability and its immediate enablers such as supply chain management, regulatory aspects, product quality and use of falsified and substandard products. Out of the 19 diagnostic frames we identified, these five frames accounted for 63 out of the 107 mentions (59% of the problems mentioned). Consequently, the prognostic frame is also dominated by interventions or policy solutions to address those problems, with 93 (44%) out of 210 frames associated with the solutions aimed at addressing these problems. This is in contrast to the global discourse where inadequate access to medicines is dominantly attributed to unaffordability that is driven by high prices and over-reliance on direct payment methods [56]. On the other hand, we found that aspects related to the financing and affordability of medicines; which are  the  core of UHC [4], were generally out -of -frame. For example, unaffordability was only mentioned as a problem in three out of the 107 diagnostic frames; accounting for 3% coverage as a problem with minimum attention to its key determinants such as the mode of financing and uneven income distribution amongst the population. A similar pattern is mirrored in the prognostic frame where interventions aimed at improving affordability such as price regulation are given scant attention. We posit that by heavily focusing on availability at the expense of affordability, there is a direct adverse implication on UHC for two main reasons. First, when medicines are available but not affordable, citizens can either forgo essential treatment or incur catastrophic and impoverishing expenditure to access them [56]. Second, peripheral attention to affordability negates the guiding pillar of equity under UHC. This is because there are particular higher-need groups such as chronic patients that are disproportionately affected by medicine unaffordability compared to the rest of the populations or those with acute needs [57]. The other out of frame, yet important issue is in relation to the problem of corruption and inefficiency in the pharmaceutical sector. None of the NSPs mentioned corruption as a problem in the pharmaceutical sector nor steps to address it although it is widely recognized as a major impediment to achieving UHC in Africa [49, 50]. In relation to motivational framing, UHC and its foundational aspects such as equity, quality and efficiency are dominantly in-frame and there is near convergence in all NSPs.

### Drivers for out-frame and in-frame issues

We posit that the biased framing towards availability is driven by an interplay of both technical and political factors. Regarding technical aspects, generally there is more data on availability indicators than affordability indicators. In turn, the diagnostic and prognostic framing of availability is backed up by credible indicators that show the severity of the problem and the effectiveness of  proposed interventions can be objetively measured over time  . For example, all the NSPs had baseline indicators on availability either with a positive frame of stock availability or negative frame in terms of stock out rates whilst there is paucity of affordability indicators such as  price information-a problem explicitly mentioned in the Nigeria NSP. Since problems that are easily measured are more likely to gain political support than those that are not [58], lack of data on affordability (the severity of the problem and effect of interventions) and the methodological challenges in measuring it [57] potentially weakens the generation of political prioritization to the problem compared to availability. In summary, whilst availability data can be used to portray a crisis in access to medicines to elicit immediate attention, there is no reliable data to portray affordability in the same crisis-laden  policy frame. The existence of global level data on unaffordability may also explain why the global diagnostic frames regard it as a crisis whilst country level framing rarely characterizes it in that manner.

As mentioned earlier, despite the conceptual and scholarly faming of corruption as a major problem for the pharmaceutical sector, the issue is evidently out of the diagnostic and prognostic frames in all the NSPs we analyzed. This is despite that accountability and transparency are emphasized as key motivational frames. This misalignment can be attributed to the political sensitivity of the issue and lack of credible indicators to measure it [51]. The other problem that is not given appropriate attention is the inherent inefficiencies in pharmaceutical procurement. In line with framing theory, under-recognition of this problem hinders countries from invoking the relevant prognostic frame. For example, although pharmaceutical expenditure can account for 25–67% of the total expenditure on health [59] and ‘lack of funds’ is often cited as a major cause for poor medicine accessibility, some viable measures to reduce prices such as price regulation and regional pooled procurement were not visibly promoted in the NSPs. Often, the diagnostic framing of inefficiency is often attributed to supply chain management bottlenecks with attached prognostic framing biased towards improving Logistics Management Information Systems and the structural design of the supply chain systems. From an issue prioritization perspective, the framing of supply chain as a major source of inefficiency might not be a true reflection of the higher magnitude of the problem compared to other forms of inefficiencies. Instead, it might be driven by the fact that there has been investments in data systems that show the severity of the problem to policy makers. In framing theory, this manifestation is related to the phenomenon of ‘fluency’ in bounded rationality where policy makers value, or pay more attention to, the things with which they are most familiar and can process more easily, though they may not be the most impotant,  and the approach may not be  optimal [60, 61].

Although we have demonstrated issues that are out of frame at national level, we also found some aspects that are prominent at national level but cautiously accepted at global level. We found traditional medicine to belong to this category. Although WHO recognizes the role of traditional medicine in the context of UHC, it highlights several risks associated with the practice and warns that its use could hinder the uptake of conventional treatments [62]. Thus, although WHO encourages countries to harness traditional medicine, from a framing perspective, its use is also de-mobilized in favor of conventional medicine at global level. On the contrary, the risks are de-emphasized at national level where traditional medicine is actively promoted from a cultural [63, 64] and public health perspective [62, 65]. The framing of access to medicines within the confines of conventional medicines in the global discourse produces some divergence in the nature and scope of Research and Development (R & D) as an anchor to improve access to medicines. Whilst at global level R & D is dominantly framed within the context of ‘missing medicines’ and the flawed patent systems that favor commercially viable products [4], in the eight African countries we studied it was framed within the motivational frame of expanding the use of traditional medicine and boosting local production. The de-emphasis of traditional medicine in the global discourse could be as a result of uncertainty in product quality [66] and regulatory challenges as highlighted in Zimbabwe. Therefore, in all the countries, the prognostic frame of promoting traditional medicine is accompanied by a greater emphasis to strengthen regulatory systems which is in concert with the global discourse [62].

### Limitations of the current framing at national level

All the NSPs reviewed mentioned UHC as a motivational frame. Ideologically, UHC is rooted in the principles of equity and its accompanying orientations such as egalitarianism, inclusivity and human rights [67]. In the context of UHC and equity, the framing of access to essential medicines does not imply only availability, instead the financial means of accessing those medicines and its consequences are equally important in line with the tenets of financial protection. Whilst the diagnostic and prognostic framing at country level endeavors to tackle the main problems in relation to ATM in the context of UHC, the are overarching limitations that warrant attention. The first limitation is the lack of sufficient attention to the complex interconnections between ATM and other health system components, particularly health financing. As described earlier, this has skewed ATM towards availability with minimal attention towards affordability and a disconnect between the over-reliance on OOP and lack of access to medicines.

The second limitation is the lack of an inter-sectoral framing to addressing problems of inadequate access to essential medicines. Despite that the provision of essential medicines is increasingly through pluralistic systems that involve a mix of both public and private players, the framing of ATM is dominantly through the public sector lens. This public-sector oriented framing presents important limitations in the context of UHC. The first one is that since availability of medicines is better in the private sector compared to the public sector [68], citizens are turning to the private sector, to access the medicines, often at prices way above international reference prices [15]. This is worth policy attention considering that expenditure on medicines is the highest driver of catastrophic expenditure and the leading barrier to UHC [69].

The other limitation is the dominance of framing that focuses on the structure of the pharmaceutical sector in terms of its organization and functions. Unlike framing for other areas such as human resources for health and disease conditions where the description of the problems is backed up by objective indicators, ATM problems and progress is mainly framed qualitatively majoring on the structural description of the pharmaceutical sector (the regulatory system, supply chain design, the process of selecting medicines and progress in revision of standard treatment guidelines). Although these are useful indicators, the dominance of this structural description is not reflective of the evolution of essential medicines concept over time. In essence, the framing of ATM is still biased towards the first era of the essential medicines concept where the motivational frame was to develop essential medicines policies despite that  the provision of medicines has evolved in line with emerging concepts such as health systems strengthening and UHC [4]. The implication of this non-evolutionary framing is that although structural indicators demonstrate the existence of a policies, this does not translate to equitable access to medicines and their rational use which is the ultimate goal in the context of UHC [4]. This limitation warants attention because as stated for the HIV/AIDS treatments, frame evolvement matters, in particular frame extensions where the boundaries of the proposed frame is strategically widened to include or encompass the views, interests, or sentiments of targeted groups [33].

Overall, this study highlights that motivational framing matters a great deal in terms of mobilizing effort for policy action. Specifically, UHC provides a very strong motivational frame to promote ATM. However, the study highlights important mismatch between problems with essential medicines as objectively identified in global discourse under UHC and the way the issue is framed in policy and practice at country level. In the eight countries we analyzed, by over-emphasizing availability at the expense of affordability, the motivational frame around UHC appeared to be largely aspirational without a comprehensive prognostic approach that can effectively address the problem of unadequate access to essential medicines. The reasons for this could be manifold, but we postulate the politics of transnational policy diffusion to be at the heart of it. Whilst transnational actors such as the WHO provide the much needed normative guidelines to shape policies, there is emphasis on how governments should strengthen technical determinants of ATM to progress towards globally conceived motivational frames such as UHC. This characterization rests on the notion of ‘policy convergence’ or the process by which knowledge about policies conceived in one jurisdiction influences policies in another jurisdiction [70]. Fundamentally, it assumes that once a country ‘adopts’ a global norm or ‘commits’ to an idea—in this case UHC- there would be synergistic action to align with that norm.This overly technocratic framing largely ignores the politics of transnational policy diffusion or how countries “mediate, filter, and refract the efforts by transnational actors and alliances to influence policies in the various issue-areas’ [71]. In the discourse of policy transfer for ATM, the dominance of the availability frame without addressing affordability despite the intricate connection between the two could be driven by the differential political risks associated with the two instruments as determinants of ATM. Generally, affordability requires fiscal commitment and a major re-design of national health financing arrangements which is fundamentally a redistributive process. In general terms, redistributive policies have political consequences and are inherently much more difficult to implement than non-redistributive policies [72]. For example, the inordinate delay to roll-out ARVs in South Africa has been attributed to the government’s instrumental calculation of the perceived economic, socio-political, and national security threats associated with the policy [73].

## Conclusion

Framing theory is applicable and useful in analyzing access to medicine aspects in the WHO African Region. We conclude that there is a mismatch between problems with essential medicines as objectively identified in global discourse and the way the issue is framed in policy and practice at country level. Whilst UHC is a strong motivational frame for guiding country level policies for ATM, it is not sufficient to drive policy action from problems to solutions at country level. Actors that actively promote a UHC oriented approach towards ATM such as UN agencies, bilateral organizations, multilateral financiers, policy makers and civic society organizations need to acknowledge such an inherent limitation and direct efforts in understanding the structural barriers for reform. Such an understanding is critical for actors to strategically frame technical solutions in a way that resonates with contextual factors to improve the implementation feasibility of reforms aimed at advancing ATM. Therefore, further in-depth country studies are needed to understand the politics of policy diffusion including, but not limited to, how the interaction of actor power, ideas, institutions and interests mediate what eventually becomes policy priority at country level in relation to ATM and its implication on UHC.

## Supplementary Information

**Additional file 1.** Details of the analyzed documents.

**Additional file 2.** Motivational framing by country.

## Data Availability

The datasets analyzed during the current study are available as supplementary files. The Zimbabwe NSP which was not yet available on the internet at the time of publication is available from the authors upon request.
