# Supplementary material for: Framing access to essential medicines in the context of Universal Health Coverage: a critical analysis of health sector strategic plans from eight countries in the WHO African region
Source: BMC Health Serv Res. 2022 Nov 22;22:1390. doi: 10.1186/s12913-022-08791-9 (PMC9682662; doi:10.1186/s12913-022-08791-9)
Supplement: Supplementary file 1 — Additional file 1. Details of the analyzed documents. [file 12913_2022_8791_MOESM1_ESM.docx]

**Additional file 1: Details of the analyzed documents**

| **Country** | **Document title** | **Year** | **URL** |
| --- | --- | --- | --- |
| Cameron | Health sector strategy | 2016-2027 | <https://www.minsante.cm/site/?q=en/content/health-sector-strategy-2016-2027-0> |
| Kenya | Kenya Health Sector  Strategic Plan | 2018-2023 | <https://www.health.go.ke/wp-content/uploads/2020/11/Kenya-Health-Sector-Strategic-Plan-2018-231.pdf> |
| Nigeria | Second national strategic health development plan | 2018-2022 | <https://www.health.gov.ng/doc/NSHDP%20II%20Final.pdf> |
| Rwanda | Fourth health sector strategic plan | 2018-2024 | <https://www.medbox.org/document/rwanda-fourth-health-sector-strategic-plan-july-2018-june-2024> |
| South Africa | Strategic plan | 2020-2025 | <https://www.health.gov.za/wp-content/uploads/2020/11/depthealthstrategicplanfinal2020-21to2024-25-1.pdf> |
| Tanzania | Health sector strategic plan | 2021-2026 | <https://www.prb.org/wp-content/uploads/2020/06/Tanzania-Health-Sector-Strategic-Plan-IV-2015-2020-1-4.pdf> |
| Zambia | Zambia national health strategic plan | 2017-2021 | <https://www.medbox.org/document/zambia-national-health-strategic-plan-2017-2021> |
| Zimbabwe | National Health strategy | 2021-2025 | Not available on the internet at the time of data analysis. Obtained through Authors’ own network |
