# Supplementary material for: Framing access to essential medicines in the context of Universal Health Coverage: a critical analysis of health sector strategic plans from eight countries in the WHO African region
Source: BMC Health Serv Res. 2022 Nov 22;22:1390. doi: 10.1186/s12913-022-08791-9 (PMC9682662; doi:10.1186/s12913-022-08791-9)
Supplement: Supplementary file 2 — Additional file 2. Motivational framing by country. [file 12913_2022_8791_MOESM2_ESM.docx]

**Additional file 2: Motivational framing by country**

|  | **Motivational framing** | |
| --- | --- | --- |
| **Country** | **Vision /Mission/Goals** | **Guiding principles** |
| Cameron | UHC , global access to quality health services for all the social strata by 2035 | Equity, quality, community participation, governance, performance based management, health partnership, devolution & centralization, participation and accountability, optimal management of health information, national solidarity, shared responsibility and social justice |
| Kenya | UHC, providing UHC under the “Big Four” agenda, a healthy, productive and globally competitive nation. | Equity, a people-centred approach, participatory approach, a multisectoral approach, efficiency and social accountability |
| Zimbabwe | UHC, vision 2030 that seeks to transform Zimbabwe into a middle income economy | Equity, quality, efficiency, confidentiality, professionalism, partnerships and multi-sectoral collaboration |
| Nigeria | UHC, to ensure that the Nigerian populace have universal access to comprehensive health care | Accountability and transparency, quality of care, ethics and respect for human rights, accessibility, affordability and acceptability, equity and gender sensitivity, community engagement, teamwork and industrial harmony, innovation, alignment and harmonization, partnership and collaboration, sustainability and resilience |
| Rwanda | UHC, Vision 2050 (“The Rwanda We Want”), which will transform Rwanda into a high-income country by 2050, SDGs | Affordability, quality |
| South Africa | UHC, a long and healthy life for all South Africans | Consultation, service standards, access, courtesy, information, openness and transparency, redress, value for money |
| Tanzania | UHC, a healthy and prosperous society that contributes fully to the development of individuals and the nation | Equity, gender, social determinants of health |
| Zambia | UHC, national vision 2030 which expresses the Zambian people’s aspiration “to become a prosperous middle-income nation by 2030 | Equity, solidarity, social justice, multi-sectoral action, community participation and unconditional enjoyment of health as a human right by all |
